# Preparation of cardan joint by selective laser sintering and properties of polyamide-12 and portland cement powder mixture

**DOI:** 10.1371/journal.pone.0328266

**Published:** 2025-08-01

**Authors:** Saleh Ahmed Aldahash

**Affiliations:** Department of Mechanical and Industrial Engineering, College of Engineering, Majmaah University, Al-Majmaah, Kingdom of Saudi Arabia; Bina Nusantara University, INDONESIA

## Abstract

A homogeneous mixture of polyamide-12 (PA-12) and Portland cement (PC) powder material was prepared using the Sinterit sifter. Many techniques were used to detect morphological and functional properties. The scanning electron microscope confirms the proper mixing of both components. Thermal testing indicates that at 453.15 K, the change in molecular weight occurred more rapidly. The results of differential scanning calorimetry confirm that as the molecular weight increases, the glass transition temperature and melting temperature increase while the crystallization temperature of the material decreases. According to the results, a mixture of PA-12 and PC powder exhibits excellent mechanical properties, making it suitable for various applications. Thermally, mechanically, and chemically, they are often the best choice in the most demanding applications that require both high mechanical strength and durability at high temperatures. Due to this, polyamide powders and cement mixtures with narrow particle sizes show promise for additive manufacturing and selective laser sintering. This study focuses on developing a new mixture material by combining cement additives with PA-12 to reduce costs and enhance the mechanical properties of sintered specimens (cardan joints). The blended material will cost less than pure PA-12 because cement is significantly cheaper than PA-12.

## Introduction

In practically all industrial and service sectors, nanotechnology can be applied to science, engineering, and technology at the nanoscale [1]. The current research on polymer nanocomposites (PNCs) within the field of nanotechnology is considered one of its most popular areas, encompassing a wide range of topics. PNCs undoubtedly make significant contributions to the advancement of nanomaterials and exhibit unique physicochemical properties that are not available through the actions alone of their components. There has been a rise in industrial interest in PNCs over the past few years [2–6]. Due to the combination of the polymer continuous phase with the nanoparticle discontinuous phase in PNCs, these devices exhibit several advantages in terms of mechanical, electric, and optical properties when compared to the same properties with individual components. In many industries, such as automotive, food and beverage packaging, energy generation and storage, as well as numerous others, PNCs have become widely used due to their diverse range of applications. In recent years, it has been widely acknowledged that polymer nano mixtures, such as polyamides (PA), polyvinyl alcohols (PVA), and polycarbonates (PC), are among the most popular and practical materials. Research indicates that polymers can be enhanced in their mechanical, thermal, electrical, and gas barrier properties by using PNCs, which utilize carbon and layered silicates [7,8]. In order to understand the behaviour of polymer nano mixtures, it is critical to consider the following information about the components of polymers: (i) their mass, (ii) their chemical structure, (iii) their semicrystalline nature, (iv) their chemical solubility, (v) their thermal stability, (vi) nanoparticle surface areas, (vii) their chemical structures, and (viii) dispersion of nanoparticles.

In engineering-grade plastics, PA (Nylon) is recognized for its exceptional strength and durability. Generally, they are semicrystalline and are resistant to both heat and chemicals. Polyamide is commonly found in various forms, including powders, blocks, rods, sheets, and even granules. PAs that occur naturally are also available as synthetic polyamides that can be synthesized artificially. In selective laser sintering (SLS), polyamide 11 and 12 powders are most commonly used at temperatures between 150°C and 185°C [9,10].

Natural polyamides, such as wool and silk, are derived from proteins, whereas synthetic polyamides, including nylons and aramids, are derived from various synthetic materials. The physical properties of abrasion resistance, chemical resistance, corrosion resistance, electromagnetic interference, and adaptability make polyamide suitable for multiple applications. There are four categories of polyamide polymers: (i) PA-6 or polyamide 6; (ii) PA-11, or polyamide 11; (iii) PA-12 or polyamide 12 and (iv) PA-66 is also known as polyamide 66 [11]. Polyamide fabric is widely used in various materials, including household and industrial products, such as utensils, carpets, curtains, food packaging, furniture, and fabrics for manufacturing applications [12]. It is possible to create PAs with only one monomer, such as amino acids or their lactam analogues, through either self-polycondensation of the monomer via ring-opening polymerization or by polycondensation of a diamine with dibasic acids [13]. Numerous finished components required for performance-oriented applications lack fire retardancy or high strength and heat resistance in PA-11 and PA-12 materials.

The paper’s purpose was to investigate the effect of cement powder on the mechanical properties of PA-12. PA-12 is characterized in this paper in several ways to highlight its different properties. PA-12 exhibits exceptional properties, making it suitable for a wide range of applications. According to the results of Horgines et al., polyamide-based waste powder can be used as lightweight aggregates, making it an interesting method for recycling [14]. Furthermore, Guler has determined that hybridized PA fibres in concrete mixes exhibit greater strength than micro- and macro-PA fibres used individually [15]. In a study, my team examined how scanning vector length and the EOV effect influence the mechanical and morphological properties of parts made from a PA-12/white cement mixture produced through SLS [16]. Additionally, my team have utilised it as an adsorbent to remove dye pollutants from coloured water [17].

Universal cardan joints are commonly used in the drivetrains of rolling mills for the production of steel, aluminium, and non-ferrous alloys. The cardan joint faces a significant problem: the output drive shaft operates at a varying speed. This inconsistency causes vibrations and accelerates wear. Vesali et al. researched the dynamics of universal joints and proposed practical methods for improving their performance by deriving the equations of motion associated with universal joints [18]. A dynamic model was used to evaluate the effects of elasticity and manufacturing discrepancies on a Cardan joint. Numerical simulations showed alignment with a GSTIFF solver [19]. The failure of a universal joint leads to an immediate halt in power transmission, potentially resulting in significant repercussions. Research on Cardan joint fatigue was conducted in the automotive column direction utilising an innovative system device [20]. A new combined fabrication process, called the 3d-PLAST manufacturing process, has recently enabled the manufacturing and characterisation of compliant Cardan joints [21].

This paper investigates the mechanical properties of a PA-12 and Portland Cement (PC) powdered mixture processed using a Sinterit sifter. Characterization shows proper mixing and preparation of PA-12 and cement. Temperature plays a crucial role in the effect of mixture materials on their thermal properties. As a result of this study, hybrid PA-12 fibres in concrete and cement mixes are more effective than micro and macro PA fibres used separately. In addition to exhibiting better mechanical properties than pure polymer, nano mixtures demonstrated improved thermal stability. The successful preparation of the cardan joint using selective laser sintering (SLS) was also conducted, and the material’s cost was calculated. The novelty and significance of this article lie in its focus on the specific applications of 3D printing techniques and the development of sophisticated polyamide 3D material in automotive applications. This is the first paper to present the use of a PA-12 and PC mixture in SLS-made cardan joints. This paper opens the door for future research in which the as-prepared Cardan joint will be investigated for its kinematic and stiffness performance as well as dynamic analysis.

## Materials and methods

### Materials

This study used a mixture of PA-12 and PC powder as the material. There is a local shop in Al-Majmaah, Saudi Arabia, where we purchased PC in black from the Saudi Cement Company. PC was made from high-quality clinker and cement with a compressive strength exceeding 40 MPa. It was created using high-quality cement with a classification ASTM C150M type l. During the production of this product, a Saudi standard of quality (SASO GSO 1917/2009) was used to ensure its quality. PC is a material that bonds solid masses or fragments together. PC is typically made from limestone or chalk and clay or shale. PC clinker is primarily composed of calcium oxide found in limestone and chalk. The oxides of silica (SiO_2_), alumina (Al_2_O_3_), and iron (Fe_2_O_3_) usually occur in large amounts in clay and shale. Consequently, Portland white cement is economically priced due to the availability of limestone and shale. The PA-12 powder we purchased has excellent mechanical properties, chemical resistance, and high-temperature compatibility (Molecular number: 7,655, Molecular weight: 21,100). It is manufactured by Sinterit and is based in Kraków, Poland. The PA-12 powder had a particle size distribution of 18–90 μm, a tensile strength of 32 MPa, and a melting point of 185 °C. The powder itself was grey. Regarding particle size distribution and shape, PA-12 is an ultrafine powder characterized by a narrow size range and nearly spherical particle shape. Additionally, it is safe for use with food and harmless to the environment.

### Fabrication of PA-12/PC nanomixture

After mixing the cement powder and PA-12, the mixture was vigorously shaken using the Sinterit sifter to prevent particle agglomeration. To obtain homogeneous powder mixtures and uniform colours, the powder mixtures were mechanically mixed for 20 minutes at a high-speed mixer, by the predetermined formulation. Clay/polymer nano mixtures must be produced using this simple method to achieve superior material properties. In the formulation of a PA-12 and PC powder material mixture, 90% PA-12 and 10% cement were used [22].

### Characterization

PerkinElmer Spectrum IR Version 10.6.1 FTIR spectroscopy was employed to analyze the structure and functionalization of a mixture of PA-12 and PC powder. With the help of JSM-6700F, JEOL, Japan, the morphological structure of polyamide-12 and PC powdered mixture was determined by scanning electron microscope (SEM). A high-resolution transmission electron microscopy (HRTEM) image of a PA-12 and PC powdered mixture was obtained to understand its morphology better. To study the thermal transition of cement and PA-12 mixture materials, Differential Scanning Calorimeters (DSC) from Mettler Toledo Thermal Analysis were used.

### SLS preparation of cardan joint

**Fig 1:**
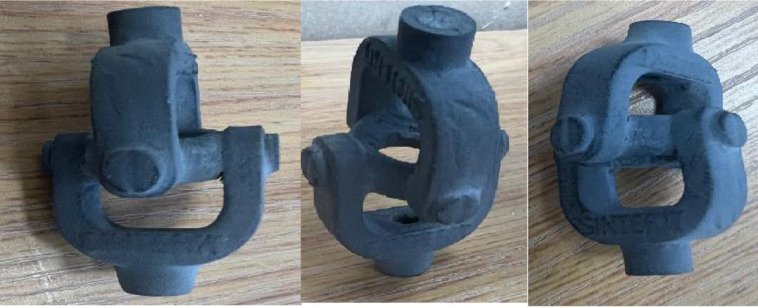
SLS made cardan joint.

**Fig 2:**
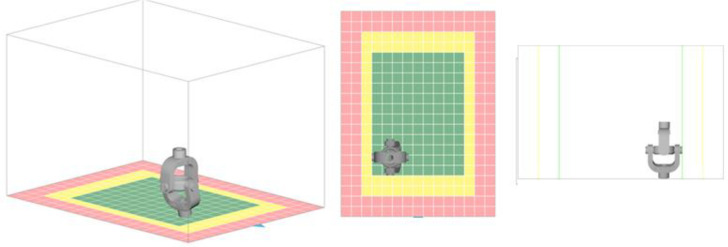
3D model of the cardan joint by Sinterit studio.

A cardan joint (U-joint) is a mechanical component typically made of metal and used to handle mechanical loads. When made from polymer materials, such as composites, the cardan joint can offer several advantages and potential limitations, such as being lightweight, corrosion-resistant, and effective. This work used selective laser sintering (SLS) to successfully make a 3D cardan joint using a PA-12/PC nano mixture, and its mechanical testing was conducted. The LISA Pro 3D SLS was purchased from Sinterit Company in Poland. It uses an IR Laser Diode with a power of 5 W at a wavelength of 808 nm. The printer’s maximum operating temperature for PA-12 is 392 °F (200 °C). Fig 1 shows the different angles of the SLS made cardan joint. The drawing was used to create a 3D model (Fig 2). The layer height of the powdered nanomixture was 0.075 mm, and in total, 3.25 L of volume of powder was used in feeding the bed. Feed bed height was 8.2 cm, total print height was 7.57 cm, and the estimated active print time was 10 h 6 m. After printing, the cardan joint was cleaned using a Sinterit sandblaster.

## Results and discussion

### SEM

**Fig 3:**
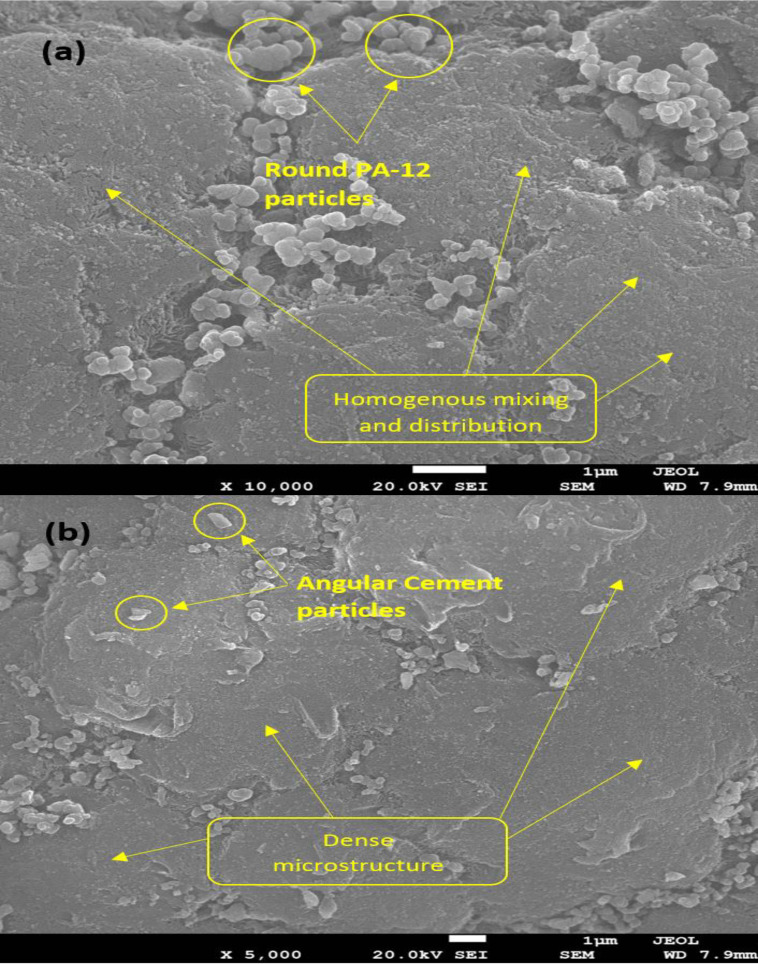
3a and 3b show SEM images of the PA-12 and PC powder mixture.

Fig 3a and 3b show SEM images of the PA-12 and PC powdered mixture, demonstrating the proper mixing of PC and PA-12. Soft, round-shaped PA-12 particles are thoroughly distributed on the PC particles in clusters. PA-12 particles can also be observed penetrating the pores of PC particles. The surface of PA-12 and PC powdered mixture particles is smooth and porous, with regular shapes and gaps.

### EDX

**Fig 4:**
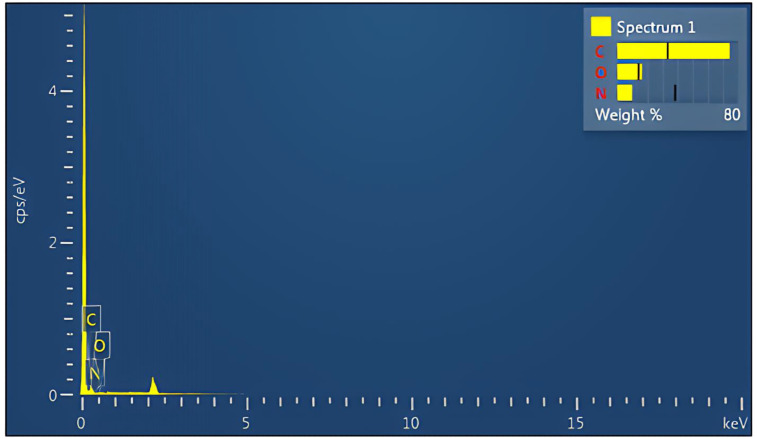
EDX analysis of the powder mixture [17].

The EDX elemental analysis of the PA-12/PC nanomixture shows that carbon is the dominant component, indicated by a prominent peak. The analysis reveals a composition of 74% carbon, 16% oxygen, and 10% nitrogen, which are evenly distributed across the powder surface (Fig 4) [17].

### FTIR

**Fig 5:**
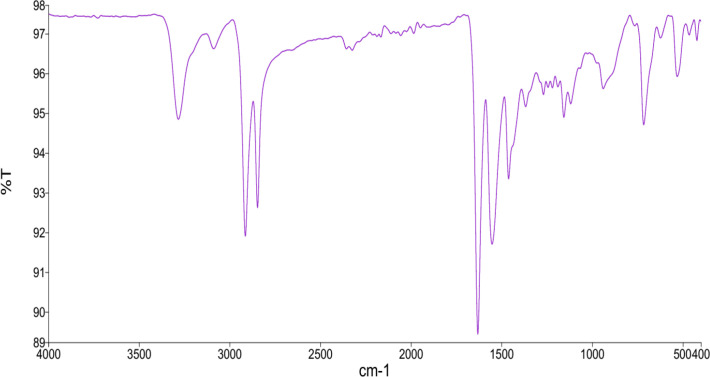
FTIR of polyamide-12 and PC powdered mixture.

FTIR Analysis, which stands for Fourier Transform Infrared Spectroscopy, is used to identify organic, polymeric, and inorganic substances. An FTIR analysis analyzes the chemical properties of test samples by scanning them with infrared light. FTIR confirmed the formation of PA-12 and cement mixtures (Fig 5). The FTIR confirmed the presence of secondary amine groups. A peak at 3040 cm^-1^ can be observed in the FTIR spectrum of the PA-12 and PC powdered mixture (Fig 5), attributed to aromatic C-H stretching. The peak at 1650 cm^-1^ is due to C = O stretching [23]. Furthermore, the peaks at 1600 and 1470 cm^-1^ are related to aromatic C = C, and the peak at 1405 cm^-1^ is associated with C-N stretching [23]. The 939, 530 and 421 cm^−1^ peaks commonly belong to the silica (Si) groups.

### TEM

**Fig 6:**
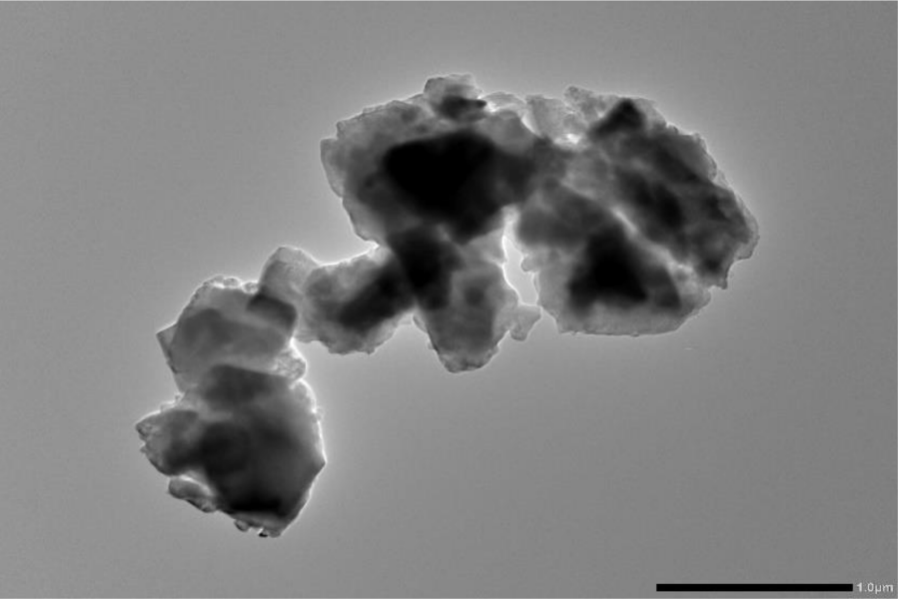
TEM of polyamide-12 and PC powdered mixture.

TEM is the method of choice for determining nanoparticle size distribution and morphology. Based on the inherent skeletal structure generated by particle accumulation, the TEM image (Fig 6) of the PA-12 and PC powdered mixture clearly shows aggregated spheroidal nanoparticles with widely distributed pores. A high dipole-dipole attraction between particles led to the mixture forming larger agglomerates [24]. No precipitation between PA-12 and cement was observed, indicating excellent compatibility [25].

### XRD

**Fig 7:**
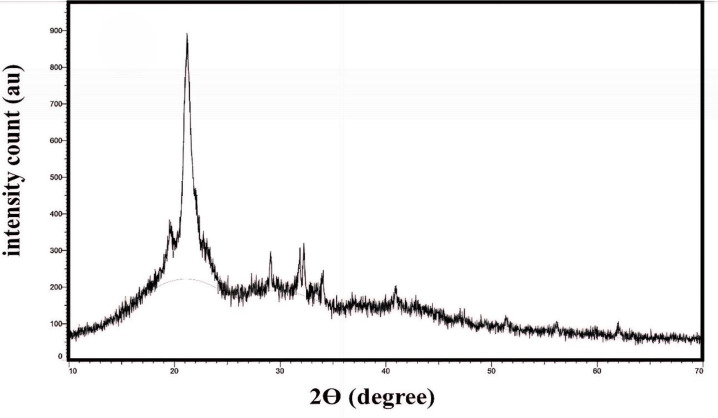
XRD analysis [17].

XRD analysis (Fig 7) reveals some crystallinity, showing numerous sharp peaks for PC and PA-12 [17]. A slight peak near 11° and a clear peak at 21.2° ~ 2θ are associated with the γ-phase, while the peak at 19.8° ~ 2θ is linked to the α-phase of PA-12. The prominent peak with an area between 20 and 25° ~ 2θ indicates that the α-phase forms during precipitation [26]. Additionally, the calcium silicate peaks suggest the presence of PC at elevated ~ 2θ values.

### Thermal properties of PA-12 and PC powdered mixture

**Fig 8:**
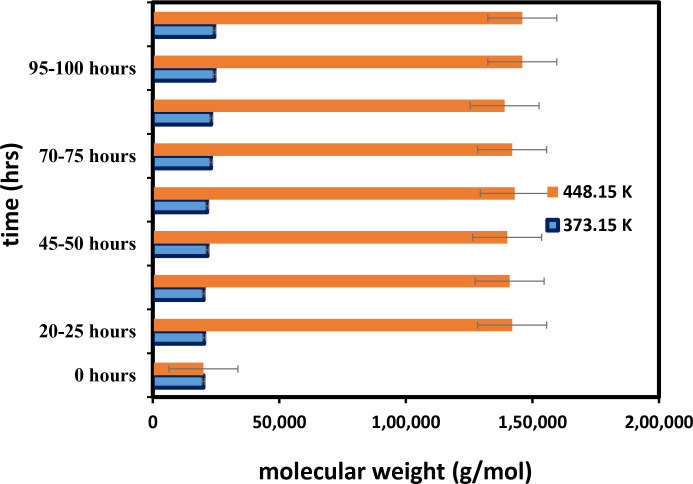
Changes in molecular weight (Mw) of a cement/PA-12 powder mixture at varying temperatures and durations.

As part of this experiment, a mixture of cement and PA-12 was heated to 373.15 and 453.15 degrees Kelvin in an oven with nitrogen. A gel permeation chromatography (GPC) test was used to determine the results (Fig 8). The figure illustrates the relationship between the molecular weight (Mw) and the heating period [27]. Based on the figure, at 373.15 K, the powder exhibited a slower increase in molecular weight, whereas at 453.15 K, the powder experienced a pronounced increase in molecular weight. At 453.15 K, the change in molecular weight occurred more rapidly. Temperature is essential in the Mw, as shown in Fig 8. At 453.15 K, during the first 25 hours of the experiment, Mw increased rapidly from 20,000 g/mol to 146,000 g/mol while the increase in Mw was slower after a 25-hour heating period. At 453.15 K, the molecular weight (Mw) increased from 20,000 g/mol to 24,300 g/mol. Since molecular size parameters are temperature dependent, the temperature dependence can affect molecular weight values. The data of this result is shown in S1 File.

**Fig 9:**
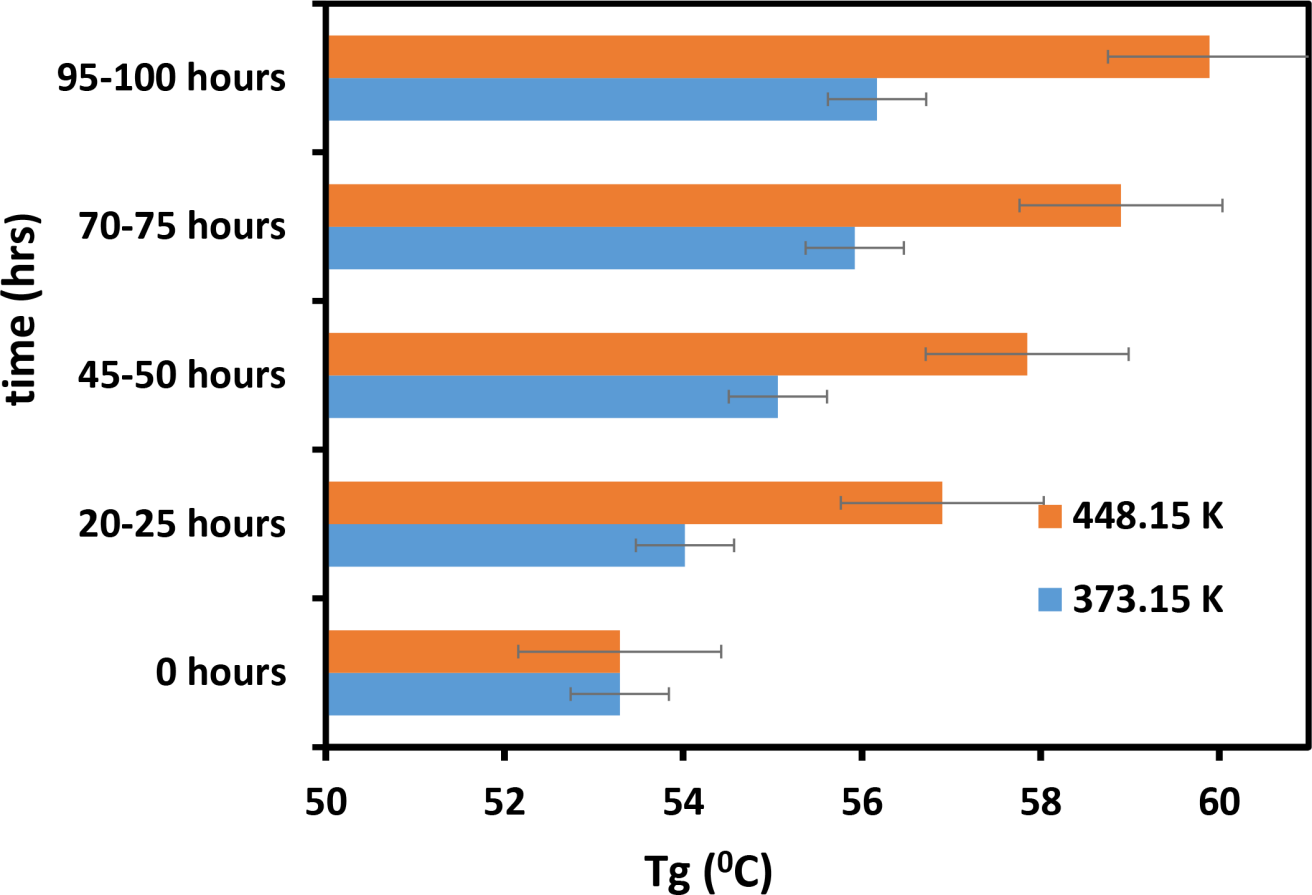
Influence of temperature and duration on the Tg of the cement/PA-12 powder mixture.

**Fig 10:**
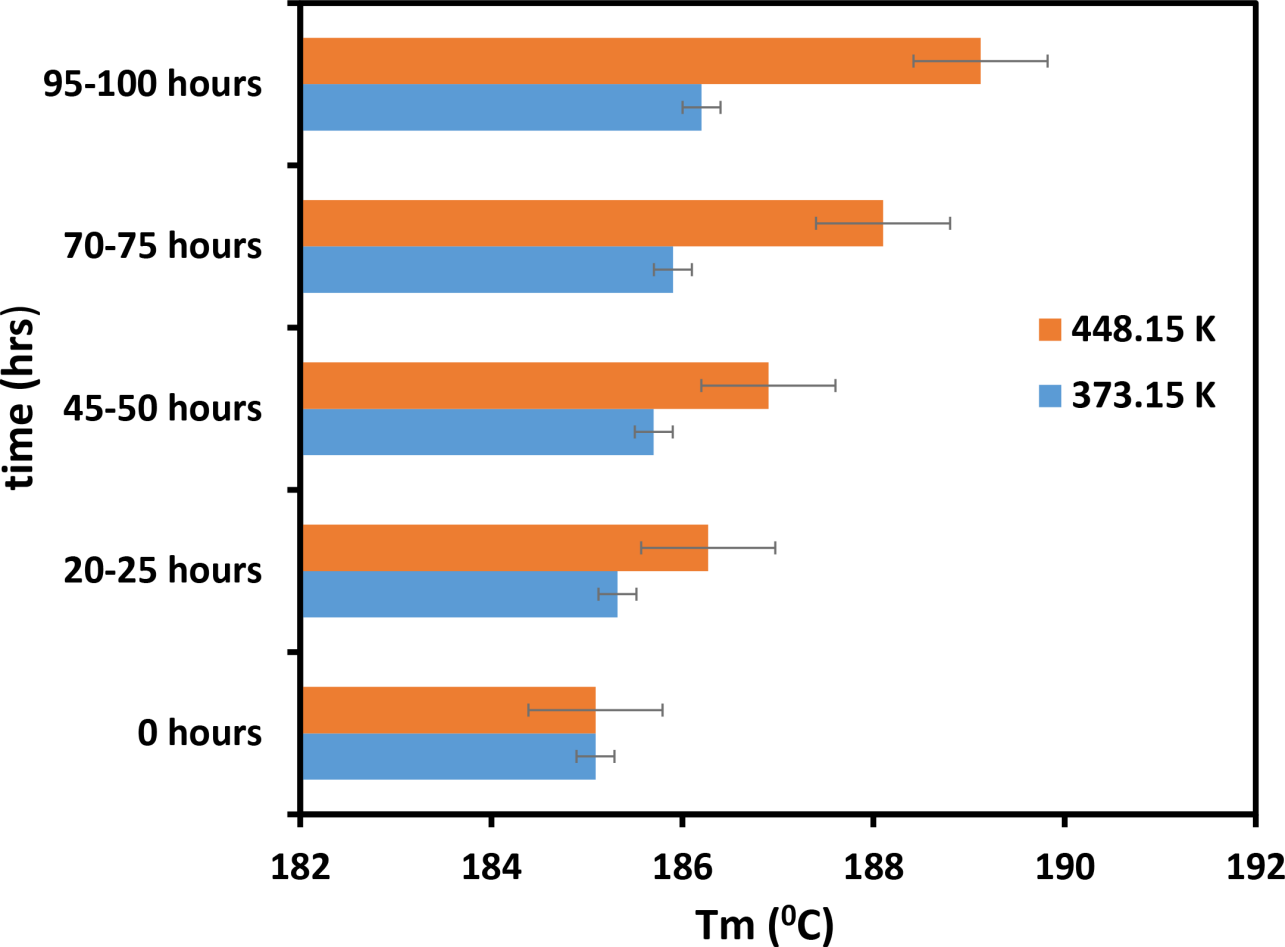
Impact of temperature and duration on the Tm of PC/PA-12 powder mixture.

**Fig 11:**
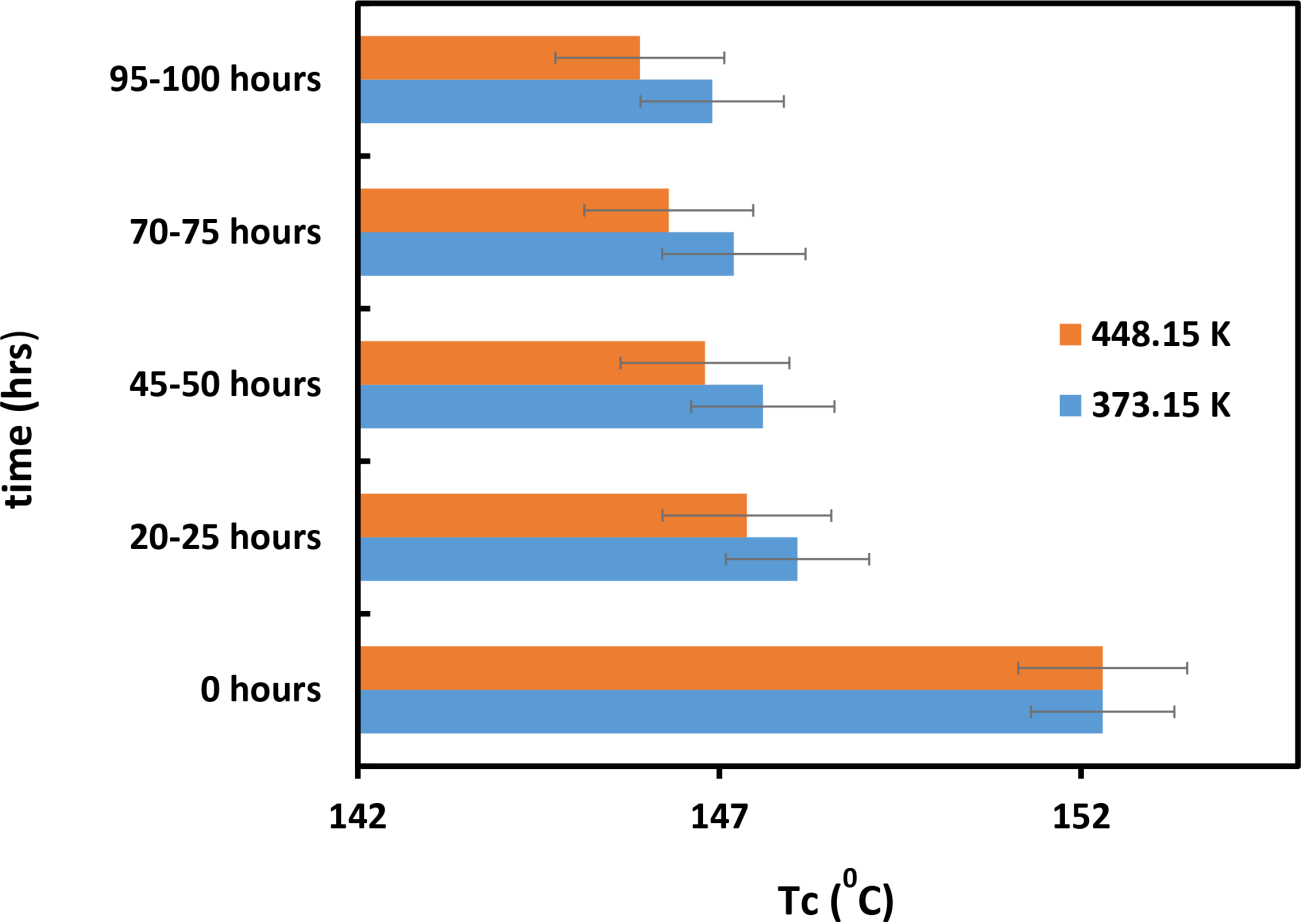
Effect of temperature and time on Tc of the mixture of PC/PA-12 powder.

Fig 9 shows that the value of Tg increased with time. Fig 9 also demonstrates that the Tg values of the powder heated to a temperature of 373.15 K are lower compared to powder heated at 453.15 K. Thus, temperature plays a crucial role in the effect of mixture materials on their thermal properties [28]. We obtained the same results with Tm, which also increased with time and had low values at high temperatures (Fig 10). Figs 9 and 10 show that at 453.15 K, both Tg and Tm increase by approximately 5°C to 7°C within 373.15 hours. During the same period, the Tg and Tm of the samples heated at 373.15 K increased slightly. In contrast, as mentioned earlier, the Tc of mixture material decreases as its molecular weight increases (Fig 11). Fig 11 shows that Tc decreases most rapidly at 453.15 K and 373.15 K within the first 25 hours, and then gradually declines. It is evident from the exact figure that Tc decreases rapidly at 453.15 K and 373.15 K in the first 25 hours. Based on these results, it can be concluded that the part bed temperature of the mixture material and its thermal properties change with temperature due to Tg, Tm, and Tc [29]. The data of these results is shown in S2 File.

### Mechanical testing

Mechanical Testing was performed following British Standards (references 527.2, 178, and 604), in compliance with American Society for Testing and Materials (ASTM) standards D0638, D0790, and D0695. When using a laser power of 3 Watts, the tensile strength, flexural yield strength, and compressive yield strength of the cardan joint (made from PA-12/cement nanomixture) were measured as 35, 49, and 38 MPa, respectively [30]. The PA-12/PC composite outperformed the basic polymer (PA-12), due to the mechanical strength and toughness of the cement matrix, which combined with the flexibility and durability of the PA-12. Additionally, the bond at the interface between the PC and PA-12 phases likely enhances stress distribution, thereby further improving the composite’s performance. Table 1 shows the complete comparative results.

**Table 1:** Mechanical Testing of PA-12/PC nanomixture.

Tensile Strength (MPa)		
**Laser Power**	**PA-12/PC composite**	**PA-12**
1	32	31.4
2	34	33.3
3	35	34.3
**Flexural Yield Strength (MPa)**		
**Laser Power**	**PA-12/PC composite**	**PA-12**
1	47	43
2	48	44
3	49	45
**Compressive Yield Strength (MPa)**		
**Laser Power**	**PA-12/PC composite**	**PA-12**
1	30	26
2	34	29
3	38	33

### Cost analysis

The material’s cost is calculated. The cardan joint was produced by SLS, for example, using 54 g of PA-12 and 6 g of PC in the preparation, resulting in 60 g of composite overall. The total cost in Saudi riyals amounts to 42.0003 (=11.20$), which includes 54 g of PA-12 costing 42 SR and 6 g of PC costing 0.0003 SR. The price for 60 g of PA-12 is 47.25 SR (=12.60$), indicating a savings of 5.24 SR (=1.24$) and thus cost-effective. A total volume of 3.25 L of powder was actually used to feed the SLS bed, costing approximately 2362.50 SR (=629.86$). This results in a savings of nearly 250 SR (=66.65$), corresponding to a 20% reduction in the price of the PA-12 bottle (2 kg, sinterit). Therefore, when preparing large polymer products, using PA-12/PC composite is a cost-effective choice.

## Conclusion

This paper investigates the mechanical properties of a PA-12 and PC powdered mixture processed using a Sinterit sifter. Characterization shows proper mixing and preparation of PA-12 and cement. Temperature plays a crucial role in the effect of mixture materials on their thermal properties. Temperature is crucial in the Mw and Tg. At 373.15 K, the powder exhibited a slower increase in molecular weight, in contrast to the pronounced increase observed at 453.15 K. At 453.15 K, the change in molecular weight was faster. The value of Tg increased with time. Alternatively, Tc decreases as its molecular weight increases. It can be concluded that the part bed temperature of the mixture material and its thermal properties change with temperature due to Tg, Tm, and Tc. As a result of this study, several industries, including manufacturing, 3D printing, and automotive, can successfully utilize a PA-12 and PC powdered mixture. Currently, SLS is the most advanced additive manufacturing technology for constructing industrial parts using polyamide. As a result of this study, hybrid PA-12 fibres in concrete and cement mixes are more effective than micro- and macro-PA fibres used separately. In addition to exhibiting better mechanical properties than pure polymer, nano mixtures demonstrated improved thermal stability. The total cost in Saudi riyals amounts to 42.0003, which includes 54 g of PA-12 costing 42 SR and 6 g of PC costing 0.0003 SR.

## Supporting information

S1 File Full dataset of the Fig 8. (XLSX)

S2 File Full dataset of the Figs 9–11. (XLSX)
